# Leptin, Both Bad and Good Actor in Cancer

**DOI:** 10.3390/biom11060913

**Published:** 2021-06-20

**Authors:** Carlos Jiménez-Cortegana, Ana López-Saavedra, Flora Sánchez-Jiménez, Antonio Pérez-Pérez, Jesús Castiñeiras, Juan A. Virizuela-Echaburu, Luis de la Cruz-Merino, Víctor Sánchez-Margalet

**Affiliations:** 1 Department of Medical Biochemistry and Molecular Biology, and Immunology, School of Medicine, Virgen Macarena University Hospital, University of Seville, 41009 Seville, Spain; cjcortegana@us.es (C.J.-C.); analopsaa@gmail.com (A.L.-S.); aerolazure@gmail.com (F.S.-J.); antonioresi@us.es (A.P.-P.); 2 Urology Service, Virgen Macarena University Hospital, University of Seville, 41009 Sevilla, Spain; uro-cast@us.es; 3 Medical Oncology Service, Virgen Macarena University Hospital, University of Seville, 41009 Sevilla, Spain; javirizuela@seom.com (J.A.V.-E.); ldelacruzmerino@gmail.com (L.d.l.C.-M.)

**Keywords:** leptin, obesity, inflammation, cancer, immune system, immunotherapy

## Abstract

Leptin is an important regulator of basal metabolism and food intake, with a pivotal role in obesity. Leptin exerts many different actions on various tissues and systems, including cancer, and is considered as a linkage between metabolism and the immune system. During the last decades, obesity and leptin have been associated with the initiation, proliferation and progression of many types of cancer. Obesity is also linked with complications and mortality, irrespective of the therapy used, affecting clinical outcomes. However, some evidence has suggested its beneficial role, called the “obesity paradox”, and the possible antitumoral role of leptin. Recent data regarding the immunotherapy of cancer have revealed that overweight leads to a more effective response and leptin may probably be involved in this beneficial process. Since leptin is a positive modulator of both the innate and the adaptive immune system, it may contribute to the increased immune response stimulated by immunotherapy in cancer patients and may be proposed as a good actor in cancer. Our purpose is to review this dual role of leptin in cancer, as well as trying to clarify the future perspectives of this adipokine, which further highlights its importance as a cornerstone of the immunometabolism in oncology.

## 1. Introduction

Leptin is a non-glycosylated hormone consisting of 167 amino acids whose existence was predicted for the first time in leptin deficient (ob/ob) and leptin receptor deficient (db/db) mice [1,2] and later described as the product of the *obese (Ob)* gene [3]. In vertebrates, leptin structures show differences in their primary amino acid sequences, but secondary and tertiary structures are similar [4], being alike to the long-chain helical cytokine family, which includes interleukin (IL) 6, IL-11, IL-12, G-CSF or oncostatin M, among others [5].

Leptin is mainly expressed in adipose tissue, but it has also been found in other tissues, such as the gastrointestinal system, the brain, or muscles [6]. In physiological conditions, leptin expression is regulated by cortisol [7], insulin [8], and IL-1b [9] during inflammation, and is an essential part of the healing process since it restores both physiological functions and homeostasis. Leptin plays a key role in inflammation due to a huge variety of metabolic effects, e.g., it increases both fatty acid oxidation [10] and glucose uptake [11]. However, inflammation sometimes takes a long period of time and could downregulate immune system functions, producing homeostatic changes and chronic pathological states, such as chronic inflammation [12]. In this context, leptin has a proinflammatory capacity, not only with a key role in obesity and food intake [13,14,15], but also in neuroendocrine regulation [16], reproduction [17,18] and diseases such as rheumatoid arthritis [19] and other autoimmune diseases [20].

Furthermore, leptin is well known to play a protumoral role, since it promotes angiogenesis, the proliferation, and survival of tumor cells, as well as the inhibition of apoptosis, leading to progression and metastasis [21]. In preclinical models, important findings related to both leptin and Ob-R levels have been found by using different strategies to overcome cancer, e.g., leptin receptor signaling has been shown to support cell metabolism in breast cancer [22], and vitamin D was found to mitigate breast tumor growth and dropped leptin levels in another study [23]. Moreover, high leptin and resistin levels impaired the therapeutic effects of dacarbazine in melanoma and their reduction improved the drug efficacy [24], which supports the importance and influence of leptin in obesity-associated conditions [25].

Conversely, leptin has been shown to reverse the immunosuppressive effects of acute starvation in mice [26]. In line with this, novel aspects have been reported in cancer. For example, leptin had antitumoral functions in human pancreatic cancer cell lines [27]. Recently, obese patients have been shown to obtain better responses to cancer immunotherapies [28,29,30], that may be related to leptin levels. For those reasons, the purpose of this article is to review the available literature concerning the relationship between leptin and chronic inflammation in cancer and the role of leptin as both a bad and good actor in the disease to better understand the possible dual effect of this hormone.

## 2. Literature Search

This literature review is the result of the study and analysis of information obtained on the relationship between leptin and cancer, approaching the role of obesity, inflammation, and immunotherapies in the disease, in the last decades. The literature search was conducted using the PubMed electronic database. The descriptors employed, being collated according to the MeSH and DeCs thesauri, were: leptin, obesity, inflammation, cancer, immunotherapy, treatment, immune system. Then, different search strategies were elaborated making use of quotation marks, truncations, and the Boolean operators AND and OR. Our search term for the PubMed database was as follows: leptin AND obesity, (leptin OR obesity) AND cancer, (leptin OR obesity) AND treatment AND cancer, (leptin OR obesity) AND immunotherapy, (leptin OR obesity) AND immune system, obesity AND immune system AND immunotherapy, (leptin OR obesity) AND inflammation, immune system AND inflammation, cancer AND inflammation, (immunotherapy OR treatment) AND inflammation AND cancer. We included research articles, meta-analysis, and reviews that met our search terms, published in the last decades, and written in English. We excluded articles and reviews with irrelevant information according to the points discussed in this review, as well as books and documents.

## 3. Role of Leptin in Inflammation

### 3.1. Leptin Receptors and Leptin Signaling Pathways

Leptin presents pleiotropic effects due to the huge variety of leptin receptors (known as Ob-R or LEPR), which belong to the class I cytokine superfamily [31,32], differing from each other in the lengths of their cytoplasmic regions and named Ob-Ra, Ob-Rb, Ob-Rc, Ob-Rd, Ob-Re and Ob-Rf. Short leptin isoforms (Ob-Ra, Ob-Rc, Ob-Rd, and Ob-Rf) can bind Janus kinases (JAK) and activate other signal transduction cascades, the soluble isoform (Ob-Re) is able to regulate leptin levels in serum, and the long leptin isoform (Ob-Rb) can fully transduce activation signals into cells via JAK2/signal transducer and activator of transcription (STAT) 3, mitogen activated protein kinase (MAPK)/extracellular-signal-regulated kinase (ERK) 1/2 or phosphatidylinositol 3-kinase (PI3K)/protein kinase B (AKT) pathways [33,34]. Ob-Rd and Ob-Rf have only been described in mice and rats, respectively [35].

### 3.2. Leptin, Inflammation, and Immune System

One of the pleiotropic effects of leptin involves the immunometabolism. Adipose tissue plays an important role in both energetic balance and storage of energy and, depending on the availability of energy, can limit or promote biological responses, such as the activation of the immune system to fight against infections [36]. Adipose tissue is also involved in inflammatory conditions by releasing hormones, anti-inflammatory, and proinflammatory factors, including leptin or adiponectin [37,38]. Leptin acts as a proinflammatory mediator in obesity-associated immune-metabolic disorders, such as diabetes, cardiovascular or autoimmune diseases, and cancer [12] by regulating hematopoiesis, lymphopoiesis, and myelopoiesis [39,40] at the development, proliferation, antiapoptotic, maturation and activation levels [41,42].

Ob-R expression is found in immune cells [36], thus leptin is involved in inflammatory/immune-related processes, e.g., by stimulating the proliferation of circulating monocytes [43]. In polymorphonuclear cells, leptin inhibits apoptosis [44,45], promotes chemotaxis [46,47], and improves the expression of CD11b via monocytes by releasing TNF-α [48], as well as stimulating the production of reactive oxygen species (ROS) [49]. Specifically in eosinophiles, leptin could suppress Intercellular Adhesion Molecule 3(ICAM-3) and enhances the expression of surface markers (e.g., ICAM-1 and CD18), and inflammatory cytokines such as IL-1β, IL-6, or IL-8 [50]. Leptin also upregulates the expression of CD63 in basophils and allows the production of type 2 cytokines such as IL-4 or IL-13 [51], which play an important role in some types of cancer [47].

In mast cells, leptin also acts as a chemoattractant and provokes the generation of histamine, cysteinyl leukotrienes, CCL2 or ROS [52], as well as causing an eosinophilic inflammation through the activation of mast cell secretory activity mediated by TNF-a, CCL5 or PGD2 [53]. Moreover, leptin-deficient mast cells take part in anti-inflammatory processes [54]. On the other hand, leptin has been described as a critical regulator for NK cell development and activation [55], since its impact on the NK immunomodulatory cytotoxicity and cytokine secretion seems unclear [56,57]. Additionally, leptin leads dendritic cell (DC) differentiation and survival [58] and helps to improve both the activation and proliferation of CD4+ and CD8+ T cells [59,60], as well as promoting Th17 differentiation [61] and Th2 responses [62]. By contrast, this hormone reduces the levels of regulatory T cells (Tregs) [63,64] and induces immunosenescence in B cells, decreasing the production of antibodies [65].

## 4. Role of Inflammation in Cancer

Inflammation is a protective response for affected tissues, that consists of the recruitment of leukocytes and plasma proteins, and causes vascularization, phagocytosis of debris and pathogens, as well as the production of proinflammatory signals such as IL-6, TNF-α or IFN-γ to recruit other immune cells. Transcriptional factors, peptides, chemokines, enzymes, lipids, and coagulation factors also take part in inflammatory processes. Once the response is finished favorably, anti-inflammatory signals (IL-10, IL-37 or TGF-β, among others) are secreted to shift the activity of the immune cells [12].

Inflammation can be developed by a huge variety of diseases, including obesity, hyperglycemia, and excessive lipid accumulation, which can promote different types of cancers [25,66,67] by releasing several factors such as ROS, TNF-α, IL-1, IL-6, IL-8, COX2, iNOS, and chemokines, among many others [68]. The inflammatory process affects during all cancer stages: In the first step, tumor initiation is produced not only by mutations and epigenetic alterations, but also by inflammatory mediators (e.g., NF-κB or STAT3 signaling) that cause tumors to be clinically evident. In the next step, cancer cells will expand until progression and metastasis. During this process, inflammatory factors such as NF-κB, IL-6 or IL-17 again act and serve like growth factors for tumor promotion, shaping cell plasticity within the TME. Later, cancer cells will spread and invade other tissues, a process in which proinflammatory molecules (e.g., TNF-α, IL-1β, or IL-11) participate to drive clonal selection of the most malignant cell clones and to recruit TGF-β and immune cells such as myeloid-derived suppressor cells (MDSCs), Tregs or M2 macrophages, that suppress T cell-mediated immune responses. Adhesion molecules and integrins (e.g., VCAM-1 and ICAM-1), produced by proinflammatory cytokines, and inflammatory cells (neutrophils and monocytes) also facilitate metastasis promoting intra- and extravasation [69].

In relation to the clinical perspective, there is consistent evidence that higher BMI is associated with increased risk of cancer despite the limitations of epidemiologic studies. Considering only obesity conditions (BMI ≥ 30 kg/m^2^), relative cancer risk was increased 1.2 to 1.5 times in multiple myeloma, from 1.5 to 1.8 in colon, gastric cardia, liver, gallbladder, kidney and pancreatic cancer, 4.8 times in esophageal adenocarcinoma, and from 2.5 to 7.1 in endometrial cancer. In addition, relative risk was increased approximately 1.1 per 5 BMI units in postmenopausal breast cancer patients [70]. For these reasons, how obesity affects outcomes in cancer is a key point to identify valid doses and treatments. There are no specific recommendations for the management of obese patients. Guideline recommendations are full weight-based chemotherapy (CT) doses [71] but, due to drug cytotoxicity and obesity-related comorbidities, obese patients can also receive CT doses based on their ideal body weight [72]. However, reductions in CT doses have been reported to be associated with increased rates of disease recurrence and mortality [73]. However, obese patients commonly present lower grades of cytotoxicity using CT compared with radiotherapy (RT) [74] and have more complications and inferior outcomes [75,76,77]. RT also alters and damages subcutaneous adipose tissue, contributing to metabolic dysfunction, which could be harmful for obese cancer patients [78].

## 5. Role of Leptin as a Bad Actor in Cancer

Over the last decades, the discovery of leptin and its receptors and their relationship with obesity and inflammation has led the possibility of new research avenues to help better understand the behavior of many diseases, including cancer. Nakao et al. (1998) described for the first time the role of leptin as a growth factor in malignant diseases by identifying Ob-R in leukemic blast cells from human samples [79].

Leptin could inhibit T cell responses by modulating both tumor cells and the TME. In obese individuals, this adipokine induces macrophages into their M2 phenotype [80], which is predominant in tumor-associated macrophages (TAMs) and plays a critical role in the initiation and progression of tumors [81]. In addition, leptin indirectly improves the role of MDSCs, a heterogeneous population of immature myeloid cells accumulated in the TME due to altered hematopoiesis, which includes granulocytic (G-MDSCs) and monocytic (M-MDSCs) precursors [82]. Leptin enhances MDSC functions via IFN-γ, which in turn is produced by leptin and regulates the checkpoint inhibitor PD-L1, causing T cell apoptosis [83]. Moreover, Ob-R blockade has been shown to decrease circulating MDSCs in breast cancer 4T1 tumor-bearing mice with a high fat diet [84].

### 5.1. Major Tumors Associated with Obesity and Leptin

Breast cancer (BC) was the most common diagnosed type of cancer worldwide during 2020 and the main cause of cancer death in the female population [85]. The relationship between obesity and BC is already established due to the complications related to surgery, radiation, and chemotherapy, with worst disease-free survival (DFS) and overall survival (OS), and an increased risk for local recurrence in female obese patients compared with their normal-weight counterparts [86] and the increased risk to develop estrogen receptor (ER)-positive BC in postmenopausal women [87,88].

Pathophysiological mechanisms remain unclear, but there are many factors involved in the association between obesity and BC, such as chronic subclinical inflammation, sex hormone deregulation, insulin/IGF-1 pathways, or the secretion of different adipokines like leptin [25]. Although some studies did not found association between leptin and BC [89,90], circulating leptin or its gene expression has been widely correlated with BC risk and progression, as well as proliferation and survival of BC cells [91,92,93,94,95,96,97,98,99,100]. Besides, leptin immunostaining has been proposed as a useful technique by supporting the identification of histotype, stage, grade, lymph node involvement, relapse, and prognosis in BC [101]. Moreover, some studies have suggested that a lower adiponectin/leptin ratio could be established in female obese patients [102,103] since low levels of the adipokine adiponectin in serum have been found to be correlated with BC risk [104]. Therefore, leptin and its receptors could be promising therapeutic targets to overcome tumor immunosuppression in the disease [105,106,107].

Colorectal cancer (CRC) is also attributed to overweight and obesity [108] and available data suggest that CRC risk is positively correlated with many factors, including metabolic syndrome [109] or leptin levels [110]. Some studies reported no associations between high serum leptin levels and CRC [111,112], but the majority described the overexpression of both leptin and Ob-R correlated with tumor progression [113,114,115,116,117]. Leptin was suggested to be a prognostic factor in CRC after analyzing survival distributions by using clinicopathological variables (tumor size, lymphovascular invasion, distant metastasis, local recurrence, and tumor relapse) in positive leptin immunostaining CRC [118]. Even leptin gene variants could exhibit sex-specific associations with CRC risk [119]. Moreover, serum leptin levels have been demonstrated to decrease following colectomy, supporting the association between leptin and CRC [120], thus suggesting leptin/Ob-R as a good target in the prevention and treatment of CRC, especially for obese patients [121].

Obesity and serum leptin are also a strong risk factors for Barrett’s esophagus [122], a precursor lesion in esophageal carcinoma [123]. Leptin and Ob-R expression has been found to play carcinogenic roles [124], since its binding activates the Akt signaling pathway in esophageal cancer cells via multiple atorvastatin sensitive small GTPases, leading to the growth and antiapoptosis of cancer cells [125]. Tumor leptin expression has also been related with chemotherapy resistance in gastro-esophageal adenocarcinomas, suggesting that leptin expression is potentially useful as a predictive marker of resistance to cytotoxic chemotherapy, and a prognostic marker independent of therapy in gastro-esophageal adenocarcinoma [126].

**Figure 1:**
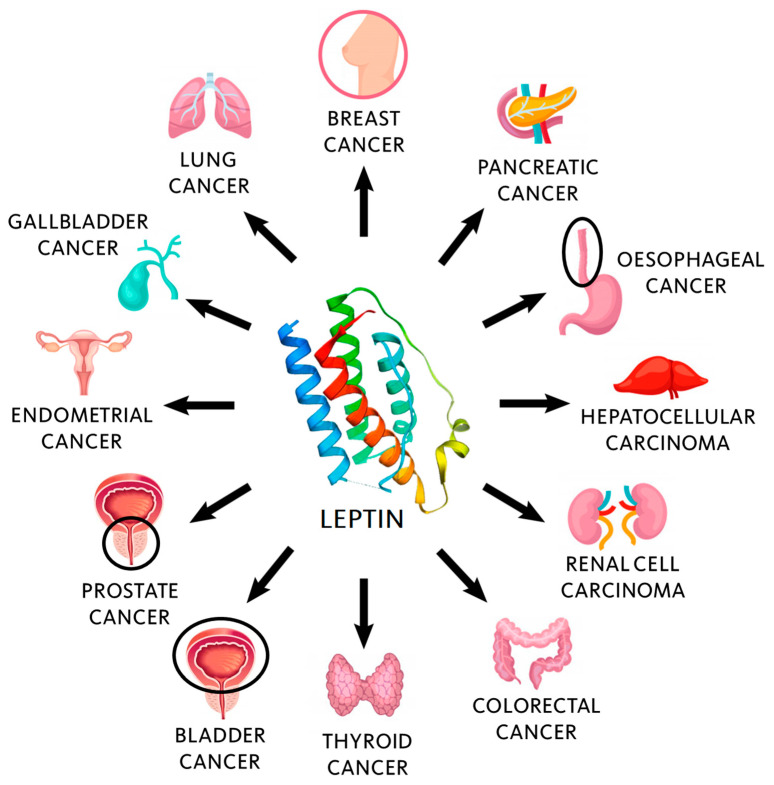
Leptin has been associated with many different types of tumors

Circulating leptin levels or leptin/Ob-R expression also promote both the survival and proliferation of tumor cells in other types of cancer. For instance, leptin increases the risk of endometrial cancer [127], independently of the obese status of patients, indicating that leptin may be involved in the endometrial carcinogenesis by other pathways [28]. In hepatocellular carcinoma (HCC) there are risk factors potentially linked to leptin (e.g., chronic hepatitis B, chronic hepatitis C, alcohol consumption, aflatoxins, and the nonalcoholic fatty liver disease) [128]. Besides, leptin takes part in angiogenesis and is considered to increase HCC risk and progression [129,130]. High leptin expression has also been found in pancreatic cancer [131,132], renal cell carcinoma [133], prostate cancer [134,135,136,137], ovarian cancer [138,139,140], bladder cancer [141,142], or gallbladder cancer [143]. Figure 1 shows some of the most common types of cancer associated with high leptin levels.

### 5.2. Mechanistic Studies

Cancer progression involves tumor initiation and growth, invasion, and metastasis. Those steps include interactions with stromal tumor components [144], such as fibroblasts, endothelial cells, immune cells, and adipocytes. The mechanisms that associate obesity and cancer as they relate to leptin are still elusive [145] and, specifically, the pathological mechanisms of leptin to be associated with poor prognosis in cancer are still unclear [21].

Leptin receptors are products of a single Ob-R [146] and its binding to leptin, explained in Section 3.1., promotes the expression of antiapoptotic proteins (e.g., X-linked inhibitor of apoptosis protein (XIAP)), systemic inflammation (e.g., IL6), angiogenic factors (e.g., VEGF) and hypoxia inducible factor-1α (HIF-1α), which promotes cancer cell survival, proliferation, and migration [147]. In line with this, leptin could play a role in vascular remodeling in hypoxia conditions through HIF-1α in human adipocytes and fibroblasts, as occurs in solid tumors [148]. Leptin can regulate angiogenesis via VEGF and IL-6, and fibroblast growth factor (FGF) 2, suppresses apoptosis through a Bcl-2-dependent mechanism and acts as a mitogen or migration factor for many different cell types, including cancer cells, as well as sustaining the recruitment of monocytes and macrophages, that allow leptin to shape the TME [149]. Moreover, leptin signaling may promote oncogenesis through the promotion of the cancer stem cell phenotype [150].

The potential role of leptin in tumor evasion and metastasis was also explained by different mechanisms that involve tumor progression and migration, including not only signaling pathways, but also cancer-associated fibroblasts, VEGF and TAMs, among many others [21]. In addition, the binding leptin/Ob-Rb has been demonstrated to induce cancer progression via extracellular matrix-cell interactions, and epithelial−mesenchymal transition, an important step during metastasis to transform epithelial cells into mesenchymal cells [151].

## 6. Role of Leptin as Good Actor in Cancer

Obesity has also been shown to play a favorable role in tumorigenesis and has been associated with better outcomes in cancer [29], suggesting the existence of what we already know as the “obesity paradox” [152,153], which is very similar to the phenomenon observed in other pathological conditions, such as cardiovascular disease [154]. The reasons for this positive association remain unknown. Most of studies analyze overweight by using BMI, although this parameter may also be increased due to the existence of protective muscle mass [155]. In addition, adipose tissue in overweight cancer patients may represent an energy store that could be used to allow a longer survival time during the disease [25]. The obesity paradox has been established in many malignancies such as lung cancer [156,157,158], renal cell carcinoma [159,160,161,162], Hodgkin and non-Hodgkin lymphoma (including aggressive B-cell lymphomas) [163,164,165], melanoma [28,166,167], acute myeloid leukemia [168], CRC [169,170,171,172,173], or HCC [174]. In this regard, a meta-analysis of 6,320,365 patients found a negative association between obesity and lung cancer, renal cell carcinoma, and melanoma, suggesting the protective role of obesity [175]. Moreover, BMI could be a useful predictive tool in combination with the anti-PD-1/PD-L1 immune checkpoint [30]. However, leptin was not measured in those studies.

The first inhibitory effect of leptin treatment in human cancer cell lines was reported in Mia-PaCa and PANC-1 pancreatic cells [27]. In addition, some studies have communicated lower serum leptin levels in premenopausal BC patients compared with healthy controls and an inverse association between serum leptin levels and BC risk [176,177]. Leptin was also correlated with better prognosis in CRC patients [178] and inhibited in vitro HCC cell growth via the p38-MAPK-dependent signaling pathway [179]. Moreover, low leptin levels have been correlated with pancreatic cancer [180,181]. Taking all these results together, we suggest the name of “leptin paradox” to explain the dual role of leptin in cancer, in a similar way to obesity. Reviewed articles in which the obesity paradox is involved in cancer and others in which leptin has been a good actor in the disease are shown in Table 1.

## 7. Clinical Applications of Leptin in Cancer

Leptin treatments have been established in some types of diseases. For example, metreleptin has been demonstrated to work in patients with lipodystrophy, hypothalamic amenorrhea, and congenital leptin deficiencies [183,184] by binding Ob-R and activating the JAK/STAT signaling pathway [185]. The combination of leptin plus amylin may be used in obese patients [186] since amylin enhances leptin signaling in both leptin-sensitive and leptin-resistant subjects [187]. Moreover, Leptin has been used as a mucosal vaccine adjuvant against *Rhodococcus equi* in mice [188].

However, leptin treatments have not yet been fully applied in cancer because the mechanisms that involve leptin in those malignancies remain unknown, especially since the dual role of leptin in carcinogenesis has been suggested. In recent years, immunotherapies have revolutionized the field of oncology [189] by enhancing the immune system functions to fight against tumor cells, thus improving clinical outcomes in different types of cancer [190]. In this regard, Rivadeneira et al. (2019) identified leptin as a potent metabolic reprogramming agent that supported antitumor responses in aggressive melanomas [182]. They demonstrated that the T cell function in tumor-bearing mice enhances antitumor response compared with an oncolytic control by designing an oncolytic virus to express leptin, as well as promoting memory responses in the oncolytic virus-induced immune infiltrate.

Leptin may improve the immunotherapic action since this adipokine has been shown to regulate both innate and adaptive responses. Leptin may increase the cytotoxicity of NK cells [191], which have been demonstrated to develop a potent cytolytic activity against tumors [192]. Leptin has also been reported to stimulate the proliferation of T cells in vitro [193], as well as activating DC [192], thus promoting the cancer-immunity cycle [194]. Moreover, leptin deficiency has been associated with low levels of blood CD4+ T cells, which were reversed by using recombinant human leptin [195]. This adipokine could also enhance the proliferation of B cells and its activation to secrete cytokines [191]. Importantly, leptin has been shown to reduce the development of Tregs [191,196], which have been associated with poor prognosis and low survival rates in many types of malignancies, including breast [197], ovarian [198], prostate [199] or lung cancer [200], among many others.

## 8. Concluding Remarks and Future Perspectives

We strongly believe in the great value of leptin as a potential biomarker not only in cancer, but also in many other diseases, since there are a wide variety of studies supporting and demonstrating this idea, including those of our research group. However, it is important to know that many limitations exist, despite the vast literature available concerning to the role of both obesity and leptin increasing cancer risk and acting as promoters of tumor development and progression. In this sense, pathophysiological mechanisms are not entirely clear, since obesity and inflammation are influenced not only by leptin, but also by many other factors [68,201,202]. Moreover, the mechanisms whereby obesity and leptin behave as good actors in cancer also remain unknown.

Nevertheless, evidence has suggested the beneficial role of obesity, which is known as the “obesity paradox”. In the same line, some studies have shown that circulating leptin levels or leptin gene expression have been negatively correlated with cancer risk or associated with better prognosis by inhibiting tumor cell growth, e.g., Rivadeneira et al. (2019) added leptin in an immunotherapic oncolytic virus in melanoma-bearing mice, with promising results [182]. The beneficial role of leptin leads us to think the existence of what we have called the “leptin paradox”. Since leptin levels are demonstrated to be dependent on the amount of adipose tissue, we believe that the “leptin paradox” could mediate the “obesity paradox”, which means that better clinical outcomes in cancer patients with higher BMI may result from the beneficial effects of leptin. Therefore, we highlight the following future steps in this field: (1) It would be interesting to analyze blood leptin levels and/or leptin/Ob-R gene expression (and not only BMI) in obese cancer patients as both predictive and prognostic clinical biomarkers, paying special attention to those patients with better outcomes, and elucidate whether better responses to immunotherapies are caused in whole or in part by leptin and its receptors, and (2) further research is also needed to fully understand the complete mechanisms by which leptin behaves as both bad and good actor, because it could lead to the design of novel therapeutic strategies to inhibit or stimulate leptin signaling, whose evaluations could be interesting in the high-risk obese populations.

## Figures and Tables

**Table 1:** Reviewed studies in which obesity plays a favorable role in cancer and those in which leptin behaves as a good actor.

Type of Cancer	Type of Study	References	Was Leptin Analyzed?	Was Leptin a Good Actor?
Breast	Humans	[176]	Yes	Yes *
	Humans	[177]	Yes	Yes
Pancreatic	Cell line	[27]	Yes	Yes
	Humans	[180]	Yes	Yes
	Humans	[181]	Yes	Yes
Melanoma	Humans	[166]	No	-
	Humans	[30]	No	-
	Mice	[182]	Yes	Yes
	Humans	[175]	No	-
Colorectal	Humans	[178]	Yes	Yes
	Humans	[169]	No	-
	Humans	[170]	No	-
	Humans	[171]	No	-
	Humans	[172]	No	-
	Humans	[173]	No	-
Renal	Humans	[159]	No	-
	Humans	[160]	No	-
	Humans	[28]	No	-
	Humans	[162]	No	-
	Humans	[30]	No	-
	Humans	[175]	No	-
Lung	Humans	[156]	No	-
	Humans	[157]	No	-
	Humans	[30]	No	-
	Humans	[158]	No	-
	Humans	[175]	No	-
Hepatocellular	Cell line	[179]	Yes	Yes
	Humans	[174]	No	-
Lymphomas	Humans	[163]	No	-
	Humans	[164]	No	-
	Humans	[165]	No	-
Myeloid leukemia	Humans	[168]	No	-
Others **	Humans	[30]	No	-

* No after adjustment of other risk factors included in the analysis. ** Types of cancer not specified in the reference.

## Data Availability

Not applicable.

## References

[1] Ingalls A.M., Dickie M.M., Snell G.D. (1950). Obese, a new mutation in the house mouse. J. Hered..

[2] Hummel K.P., Dickie M.M., Coleman D.L. (1966). Diabetes, a new mutation in the mouse. Science.

[3] Zhang Y., Proenca R., Maffei M. (1994). Positional cloning of the mouse obese gene and its human homologue. Nature.

[4] Denver R.J., Bonett R.M., Boorse G.C. (2011). Evolution of leptin structure and function. Neuroendocrinology.

[5] Zhang F., Basinski M.B., Beals J.M., Briggs S.L., Churgay L.M., Clawson D.K., DiMarchi R.D., Furman T.C., Hale J.E., Hsiung H.M. (1997). Crystal structure of the obese protein leptin-E100. Nature.

[6] Zhang Y., Chua S. (2017). Leptin Function and Regulation. Compr. Physiol..

[7] Leal-Cerro A., Soto A., Martinez M.A., Dieguez C., Casanueva F.F. (2001). Influence of cortisol status on leptin secretion. Pituitary.

[8] Paz-Filho G., Mastronardi C., Wong M.-L., Licinio J. (2012). Leptin therapy, insulin sensitivity, and glucose homeostasis. Indian J. Endocrinol. Metab..

[9] Faggioni R., Fantuzzi G., Fuller J., Dinarello C.A., Feingold K.R., Grunfeld C. (1998). IL-1 beta mediates leptin induction during inflammation. Am. J. Physiol..

[10] Sterm J.H., Rutkowski J.M., Scherer P.E. (2016). Adiponectin, Leptin, and Fatty Acids in the Maintenance of Metabolic Homeostasis through Adipose Tissue Crosstalk. Cell Metab..

[11] Meek T.H., Morton G.J. (2016). The role of leptin in diabetes: Metabolic effects. Diabetologia.

[12] Pérez-Pérez A., Sánchez-Jiménez F., Vilariño-García T., Sánchez-Margalet V. (2020). Role of leptin in inflammation and vice versa. Int. J. Mol. Sci..

[13] Farr O.M., Gavrieli A., Mantzoros C.S. (2015). Leptin applications in 2015: What have we learned about leptin and obesity?. Curr. Opin. Endocrinol. Diabetes Obes..

[14] Wasim M., Awan F.R., Najam S.S., Khan A.R., Khan H.N. (2016). Role of leptin deficiency, inefficiency, and leptin receptors in obesity. Biochem. Genet..

[15] Montserrat-de la Paz S., Pérez-Pérez A., Vilariño-García T., Jiménez-Cortegana C., Muriana F.J.G., Millán-Linares M.C., Sánchez-Margalet V. (2021). Nutritional modulation of leptin expression and leptin action in obesity and obesity-associated complications. J. Nutr. Biochem..

[16] Khan S.M., Hamnvik O.P.R., Brinkoetter M., Mantzoros C.S. (2012). Leptin as a modulator of neuroendocrine function in humans. Yonsei Med. J..

[17] Caprio M., Fabbrini E., Isidori A.M., Aversa A., Fabbri A. (2001). Leptin in reproduction. Trends Endocrinol. Metab..

[18] Pérez-Pérez A., Toro A., Vilariño-García T., Maymó J., Guadix P., Dueñas J.L., Fernández-Sánchez M., Varone C., Sánchez-Margalet V. (2018). Leptin action in normal and pathological pregnancies. J. Cell. Mol. Med..

[19] Tian G., Liang J.-N., Wang Z.-Y., Zhou D. (2014). Emerging role of leptin in rheumatoid arthritis. Clin. Exp. Immunol..

[20] La Cava A. (2017). Leptin in inflammation and autoimmunity. Cytokine.

[21] Ray A., Cleary M.P. (2017). The potential role of leptin in tumor evasion and metastasis. Cytokine Growth Factor Rev..

[22] Park J., Kusminski C.M., Chua S.C., Scherer P.E. (2010). Leptin receptor signaling supports cancer cell metabolism through suppression of mitochondrial respiration in vivo. Am. J. Pathol..

[23] Swami S., Krishnan A.V., Williams J., Aggarwal A., Albertelli M.A., Horst R.L., Feldman B.J., Feldman D. (2016). Vitamin D mitigates the adverse effects of obesity on breast cancer in mice. Endocr. Relat. Cancer.

[24] Malvi P., Chaube B., Singh S.V., Mohammad N., Vijayakumar M.V., Singh S., Chouhan S., Bhat M.K. (2018). Elevated circulatory levels of leptin and resistin impair therapeutic efficacy of dacarbazine in melanoma under obese state. Cancer Metab..

[25] Sánchez-Jiménez F., Pérez-Pérez A., De la Cruz-Merino L., Sánchez-Margalet V. (2019). Obesity and Breast cancer: Role of leptin. Front. Oncol..

[26] Lord G.M., Matarese G., Howard J.K., Baker R.J., Bloom S.R., Lechler R.I. (1998). Leptin modulates the T-cell immune response and reverses starvation-induced immunosuppression. Nature.

[27] Somasundar P., Yu A.K., Vona-Davis L., McFadden D.W. (2003). Differential effects of leptin on cancer in vitro. J. Surg. Res..

[28] Hayes A.J., Larkin J. (2018). BMI and outcomes in melanoma: More evidence for the obesity paradox. Lancet Oncol..

[29] Murphy W.J., Longo D.L. (2019). The surprisingly positive association between obesity and cancer immunotherapy efficacy. JAMA.

[30] Cortellini A., Bersanelli M., Buti S., Cannita K., Santini D., Perrone F., Giusti R., Tiseo M., Michiara M., Di Marino P. (2019). A multicenter study of body mass index in cancer patients treated with anti-PD-1/PD-L1 immune checkpoint inhibitors: When overweight becomes favorable. J. Immunother. Cancer.

[31] Tartaglia L.A., Dembski M., Weng X., Deng N., Culpepper J., Devos R., Richards G.J., Campfield L.A., Clark F.T., Deeds J. (1995). Identification and expression cloning of a leptin receptor OB-R. Cell.

[32] Tartaglia L.A. (1997). The leptin receptor. J. Biol. Chem..

[33] Gorska E., Popko K., Stelmaszczyk-Emmel A., Ciepiela O., Kucharska A., Wasik M. (2010). Leptin receptors. Eur. J. Med. Res..

[34] Park H.-Y., Ahima R.S. (2014). Leptin signaling. F1000Prime Rep..

[35] Chua S.C., Koutras I.K., Han L., Liu S.M., Kay J., Young S.J., Chung W.K., Leibel R.L. (1997). Fine structure of the murine leptin receptor gene: Splice site suppression is required to form two alternatively spliced transcripts. Genomics.

[36] Pérez-Pérez A., Vilariño-García T., Fernández-Riejos P., Martín-González J., Segura-Egea J.J., Sánchez-Margalet V. (2017). Role of leptin as a link between metabolism and the immune system. Cytokine Growth Factor Rev..

[37] Fantuzzi G. (2005). Adipose tissue, adipokines, and inflammation. J. Allergy Clin. Immunol..

[38] Wozniak S.E., Gee L.L., Wachtel M.S., Frezza E.E. (2009). Adipose tissue: The new endocrine organ? A review article. Dig. Dis. Sci..

[39] Lam Q.L.K., Lu L. (2007). Role of leptin in immunity. Cell. Mol. Immunol..

[40] Claycombe K., King L.E., Fraker P.J. (2008). A role for leptin in sustaining lymphopoiesis and myelopoiesis. Proc. Natl. Acad. Sci. USA.

[41] Stofkova A. (2009). Leptin and adiponectin: From energy and metabolic dysbalance to inflammation and autoimmunity. Endocr. Regul..

[42] Fernández-Riejos P., Najib S., Santos-Álvarez J., Martín-Romero C., Pérez-Pérez A., González-Yanes C., Sánchez-Margalet V. (2010). Role of leptin in the activation of immune cells. Mediat. Inflamm..

[43] Santos-Álvarez J., Goberna R., Sánchez-Margalet V. (1999). Human leptin stimulates proliferation and activation of human circulating monocytes. Cell Immunol..

[44] Bruno A., Conus S., Schmid I., Simon H.-U. (2005). Apoptotic pathways are inhibited by leptin receptor activation in neutrophils. J. Immunol..

[45] Conus S., Bruno A., Simon H.-U. (2005). Leptin is an eosinophil survival factor. J. Allergy Clin. Immunol..

[46] Cadelfie-Chezet F., Poulin A., Vasson M.P. (2003). Leptin regulates functional capacities of polymorphonuclear neutrophils. Free Radic. Res..

[47] Cadelfie-Chezet F., Poulin A., Tridon A., Sion B., Vasson M.P. (2001). Leptin: A potential regulator of polymorphonuclear neutrophil bactericidal action?. J. Leukoc. Biol..

[48] Zarkesh-Esfahani H., Pockley G.A., Wu Z., Hellewell P.G., Weetman A.P., Ross R.J.M. (2004). Leptin indirectly activates human neutrophils via induction of TNF-alpha. J. Immunol..

[49] Suzuki A., Leland P., Joshi B.H., Puri R.K. (2015). Targeting of IL-4 and IL-13 receptors for cancer therapy. Cytokine.

[50] Wong C.K., Cheung P.F.-Y., Lam C.W.K. (2007). Leptin-mediated cytokine release and migration of eosinophils: Implications for immunopathophysiology of allergic inflammation. Eur. J. Immunol..

[51] Suzukawa M., Nagase H., Ogahara I., Han K., Tashimo H., Shibui A., Koketsu R., Nakae S., Yamaguchi M., Ohta K. (2011). Leptin enhances survival and induces migration, degranulation, and cytokine synthesis of human basophils. J. Immunol..

[52] Milling S. (2019). Adipokines and the control of mast cell functions: From obesity to inflammation?. Immunology.

[53] Amorim N.R.T., Souza-Almeida G., Luna-Gomes T., Bozza P.T., Canetti C., Diaz B.L., Maya-Monteiro C.M., Bandeira-Melo C. (2020). Leptin elicits in vivo eosinophil migration and activation: Key role of mast cell-derived PGD_2_. Front. Endocrinol. (Lausanne).

[54] Zelechowska P., Brzezinska-Blaszczyk E., Wiktorska M., Rózalska S., Wawrocki S., Kozlowska E., Agier J. (2019). Adipocytokines leptin and adiponectin function as mast cell activity modulators. Immunology.

[55] Tian Z., Sun R., Wei H., Gao B. (2002). Impaired natural killer (NK) cell activity in leptin receptor deficient mice: Leptin as a critical regulator in NK cell development and activation. Biochem. Biophys. Res. Commun..

[56] Bähr I., Spielmann J., Quandt D., Kielstein H. (2020). Obesity-associated alterations of natural killer cells and immunosurveillance of cancer. Front. Immunol..

[57] Huebner L., Engeli S., Wrann C.D., Goudeva L., Laue T., Kielstein H. (2013). Human NK cell subset functions are differentially affected by adipokines. PLoS ONE.

[58] Mattioli B., Straface E., Quaranta M.G., Giordani L., Viora M. (2005). Leptin promotes differentiation and survival of human dendritic cells and licenses them for Th1 priming. J. Immunol..

[59] Martín-Romero C., Santos-Álvarez J., Goberna R., Sánchez-Margalet V. (2000). Human leptin enhances activation and proliferation of human circulating T lymphocytes. Cell Immunol..

[60] Kim S.Y., Lim J.H., Choi S.W., Kim M., Kim S.-T., Kim M.-S., Cho Y.S., Chun E., Lee K.-Y. (2010). Preferential effects of leptin on CD4 T cells in central and peripheral immune system are critically linked to the expression of leptin receptor. Biochem. Biophys. Res. Commun..

[61] Reis B.S., Lee K., Fanok M.H., Mascarague C., Amoury M., Cohn L.B., Rogoz A., Dallner O.S., Moraes-Vieira P.M., Domingos A.I. (2015). Leptin receptor signaling in T cells is required for Th17 differentiation. J. Immunol..

[62] Zheng H., Zhang X., Castillo E.F., Luo Y., Liu M., Yang X.O. (2016). Leptin enhances Th2 and ILC2 responses in allergic airway disease. J. Biol. Chem..

[63] De Rosa V., Procaccini C., Calì G., Pirozzi G., Fontana S., Zappacosta S., La Cava A., Matarese G. (2007). A key role of leptin in the control of regulatory T cell proliferation. Immunity.

[64] Matarese G., Procaccini C., De Rosa V., Horvath T.L., La Cava A. (2010). Regulatory T cells in obesity: The leptin connection. Trends Mol. Med..

[65] Frasca D., Díaz A., Romero M., Blomberg B.B. (2020). Leptin induces immunosenescence in human B cells. Cell. Immunol..

[66] Quail D.F., Dannenberg A.J. (2019). The obese adipose tissue microenvironment in cancer development and progression. Nat. Rev. Endocrinol..

[67] Kolb R., Sutterwala F.S., Zhang W. (2016). Obesity and cancer: Inflammation bridges the two. Curr. Opin. Pharmacol..

[68] Singh N., Baby D., Rajguru J.P., Patil P.B., Thakkannavar S.S., Pujari V.B. (2019). Inflammation and cancer. Ann. Afr. Med..

[69] Greten F.R., Grivennikov S.I. (2019). Inflammation and cancer: Triggers, mechanisms, and consequences. Immunity.

[70] Lauby-Secretan B., Scoccianti C., Loomis D., Grosse Y., Bianchini F., Straif K., International Agency for Research on Cancer Handbook Working Group (2016). Body Fatness and Cancer—Viewpoint of the IARC Working Group. N. Engl. J. Med..

[71] Renehan A.G., Harvie M., Cutress R.I., Leitzmann M., Pischon T., Howell S., Howell A. (2016). How to Manage the Obese Patient with Cancer. J. Clin. Oncol..

[72] Griggs J.J., Mangu P.B., Anderson H., Balaban E.P., Digman J.J., Hryniuk W.M., Morrison V.A., Pini T.M., Runowicz C.D., Rosner G.L. (2012). Appropriate chemotherapy dosing for obese adult patients with cancer: American Society of Clinical Oncology clinical practice guideline. J. Clin. Oncol..

[73] Lyman G.H., Sparreboom A. (2013). Chemotherapy dosing in overweight and obese patients with cancer. Nat. Rev. Clin. Oncol..

[74] Ross K.H., Gogineni K., Subhedar P.D., Lin J.Y., McCullough L.E. (2019). Obesity and cancer treatment efficacy: Existing challenges and opportunities. Cancer.

[75] Lee K., Kruper L., Dieli-Conwright C.M., Mortimer J.E. (2019). The Impact of Obesity on Breast Cancer Diagnosis and Treatment. Curr. Oncol. Rep..

[76] Smyth H. (2014). Does Obesity Impact Treatment Outcome for Prostate Cancer Patients Treated with Radiotherapy: The Weighted Debate. J. Cancer Prev. Curr. Res..

[77] Moszyńska-Zielińska M., Chałubińska-Fendler J., Gottwald L., Żytko L., Bigos E., Fijuth J. (2014). Does obesity hinder radiotherapy in endometrial cancer patients? The implementation of new techniques in adjuvant radiotherapy—Focus on obese patients. Prz. Menopauzalny.

[78] Poglio S., Galvani S., Bour S., André M., Prunet-Marcassus B., Pénicaud L., Casteilla L., Cousin B. (2009). Adipose Tissue Sensitivity to Radiation Exposure. Am. J. Pathol..

[79] Nakao T., Hino M., Yamane T., Nishizawa Y., Morii H., Tatsumi N. (1998). Expression of the leptin receptor in human leukaemic blast cells. Br. J. Haematol..

[80] Acedo S.C., Gambero S., Pereira-Cunha F.G., Lorand-Metze I., Gambero A. (2013). Participation of leptin in the determination of the macrophage phenotype: An additional role in adipocyte and macrophage crosstalk. In Vitro Cell. Dev. Biol. Anim..

[81] Lin Y., Xu J., Lan H. (2019). Tumor-associated macrophages in tumor metastasis: Biological roles and clinical therapeutic applications. J. Hematol. Oncol..

[82] Gabrilovich D.I. (2017). Myeloid-derived suppressor cells. Cancer Immunol. Res..

[83] Ostrand-Rosenberg S. (2018). Myeloid Derived-Suppressor Cells: Their role in cancer and obesity. Curr. Opin. Immunol..

[84] Clements V.K., Long T., Long R., Figley C., Smith D.M.C., Ostrand-Rosenberg S. (2018). Frontline Science: High fat diet and leptin promote tumor progression by inducing myeloid-derived suppressor cells. J. Leukoc. Biol..

[85] Sung H., Ferlay J., Siegel R.L., Laversanne M., Soerjomataram I., Jemal A., Bray F. (2021). Global cancer statistics 2020: Globocan estimates of incidence and mortality worldwide for 36 cancers in 185 countries. CA Cancer J. Clin..

[86] Brown K.A. (2021). Metabolic pathways in obesity-related breast cancer. Nat Rev Endocrinol..

[87] Neuhouser M.L., Aragaki A.K., Prentice R.L., Manson J.E., Chlebowski R., Carty C.L., Ochs-Balcom H.M., Thomson C.A., Cann B.J., Tinker L.F. (2015). Overweight, obesity, and postmenopausal invasive breast cancer risk: A secondary analysis of the Women’s Health Initiative Randomized Clinical Trials. JAMA Oncol..

[88] McKenzie F., Ferrari P., Freisling H., Chajès V., Rinaldi S., de Batlle J., Dahm C.C., Overvad K., Baglietto L., Dartois L. (2015). Healthy lifestyle and risk of breast cancer among postmenopausal women in the European prospective investigation into cancer and nutrition cohort study. Int. J. Cancer.

[89] Aliustaoglu M., Bilici A., Gumus M., Colak A.T., Baloglu G., Irmak R., Seker M., Ustaalioglu B.B.O., Salman T., Sonmez B. (2010). Preoperative serum leptin levels in patients with breast cancer. Med. Oncol..

[90] Gu F., Kraft P., Rice M., Michels K.B. (2012). Leptin and leptin receptor genes in relation to premenopausal breast cancer incidence and grade in Caucasian women. Breast Cancer Res. Treat..

[91] Niu J., Jiang L., Guo W., Shao L., Liu Y., Wang L. (2013). The association between leptin level and breast cancer: A meta-analysis. PLoS ONE.

[92] Pan H., Deng L.L., Cui J.Q., Shi L., Yang Y.C., Luo J.H., Qin D., Wang L. (2018). Association between serum leptin levels and breast cancer risk: An updated systematic review and meta-analysis. Medicine.

[93] Gui Y., Pan Q., Chen X., Xu S., Luo X., Chen L. (2017). The association between obesity related adipokines and risk of breast cancer: A meta analysis. Oncotarget.

[94] Garofalo C., Koda M., Cascio S., Sulkowska M., Kanczuga Koda L., Golaszewska J., Russo A., Sulkowki S., Surmacz E. (2006). Increased expression of leptin and the leptin receptor as a marker of breast cancer progression: Possible role of obesity-related stimuli. Clin. Cancer Res..

[95] Al Awadhi S.A., Al Khaldi R.M., Al Rammah T., Kapila K., Mojjminiyi O.A. (2012). Associations of adipokines & insulin resistance with sex steroids in patients with breast cancer. Indian J. Med. Res..

[96] Romero-Figueroa M.S., Garduno-García J.J., Duarte-Mote J., Matute-González G., Gómez-Villanueva A., De la Cruz-Vargas J. (2013). Insulin and leptin levels in obese patients with and without breast cancer. Clin. Breast Cancer.

[97] Chang C.-C., Wu M.-J., Yang J.-Y., Camarillo I.G., Chang C.-J. (2015). Leptin-STAT3-G9a signaling promotes Obesity-mediated breast cancer progression. Cancer Res..

[98] El-Hussiny M.A., Atwa M.A., Rashad W.E., Shaheen D.A., Elkady N.M. (2017). Leptin receptor Q223R polymorphism in Egyptian female patients with breast cancer. Contemp. Oncol. (Pozn).

[99] Matini A.-H., Abdirad A., Omranipour R., Shahsiah R. (2015). Comparison of serum leptin levels among patients with benign or malignant breast lesions. Arch. Breast Cancer.

[100] Boothby-Shoemaker W., Benham V., Paithankar S., Shankar R., Chen B., Bernard J.J. (2020). The relationship between leptin, the leptin receptor and FGFR1 in primary human breast tumors. Cells.

[101] Khabaz M.N., Abdelrahman A., Butt N., Damnhory L., Elshal M., Aldahlawi A.M., Ashoor S., Al-Maghrabi B., Dobson P., Brown B. (2017). Immunohistochemical staining of leptin is associated with grade, stage, lymph node involvement, recurrence, and hormone receptor phenotypes in breast cancer. BMC Womens Health.

[102] Chen D.C., Chung Y.F., Yeh Y.T., Chaung H.C., Kuo F.C., Fu O.Y., Chen H.Y., Hou M.F., Yuan S.S. (2006). Serum adiponectin and leptin levels in Taiwanese breast cancer patients. Cancer Lett..

[103] Grossmann M.E., Cleary M.P. (2012). The balance between leptin and adiponectin in the control of carcinogenesis—Focus on mammary tumorigenesis. Biochimie.

[104] Miyoshi Y., Funahashi T., Kihara S., Taguchi T., Tamaki Y., Matsuzawa Y., Noguchi S. (2003). Association of serum adiponectin levels with breast cancer risk. Clin. Cancer Res..

[105] Hu X., Juneja S.C., Maihle N.J., Cleary M.P. (2002). Leptin—a growth factor in normal and malignant breast cells and for normal mammary gland development. J. Natl. Cancer Inst..

[106] Surmacz E. (2007). Obesity hormone leptin: A new target in breast cancer?. Breast Cancer Res..

[107] Guo S., Liu M., Wang G., Torroella-Kouri M., González-Pérez R.R. (2012). Oncogenic role and therapeutic target of leptin signaling in breast cancer and cancer stem cells. Biochim. Biophys. Acta.

[108] Bardou M., Barkun A.N., Martel M. (2013). Obesity and colorectal cancer. Gut.

[109] Lee J., Lee K.S., Kim H., Jeong H., Choi M.-J., Yoo H.-W., Han T.-H., Lee H. (2020). The relationship between metabolic syndrome and the incidence of colorectal cancer. Environ. Health Prev. Med..

[110] Kim H.R. (2015). Obesity-related colorectal cancer: The role of leptin. Ann. Coloproctol..

[111] Wallace A.M., Sattar N., McMillan D.C. (1998). Effect of weight loss and the inflammatory response on leptin concentrations in gastrointestinal cancer patients. Clin. Cancer Res..

[112] Arpaci F., Yilmaz M.I., Ozet A., Ayta H., Ozturk B., Komurcu S., Ozata M. (2002). Low serum leptin level in colon cancer patients without significant weight loss. Tumori J..

[113] Mhaidat N.M., Alzoubi K.H., Kubas M.A., Banihani M.N., Hamdan N., Al-Jaberi T.M. (2021). High levels of leptin and non-high molecular weight-adiponection in patients with colorectal cancer: Association with chemotherapy and common genetic polymorphisms. Biomed. Rep..

[114] Liu H., Wan D., Pan Z., Cao L., Wu X., Lu Z., Kang T. (2011). Expression and biological significant of leptin, leptin receptor, VEGF, and CD34 in colorectal carcinoma. Cell Biochem. Biophys..

[115] Vuletic M.S., Milosevic V.S., Jancic S.A., Zujovic J.T., Krstic M.S., Vukmirovic F.C. (2019). Clinical significance of leptin receptor (LEPR) and endoglin (CD105) expressions in colorectal adenocarcinoma. J. BUON.

[116] Al-Shibli S.M., Harun N., Ashour A.E., Kasmuri M.H.B.M., Mizan S. (2019). Expression of leptin and leptin receptors in colorectal cancer-an immunohistochemical study. PerrJ.

[117] Li C., Quan J., Wei R., Zhao Z., Guan X., Liu Z., Zou S., Wang X., Jiang Z. (2020). Leptin overexpression as a poor prognostic factor for colorectal cancer. Biomed. Res. Int..

[118] Al-Maghrabi J.A., Qureshi I.A., Khabaz M.N. (2018). Expression of leptin in colorectal adenocarcinoma showed significant different survival patterns associated with tumor size, lymphovascular invasion, distant metastasis, local recurrence, and relapse of disease in the western province of Saudi Arabia. Medicine (Baltimore).

[119] Chun K.A., Kocarnik J.M., Hardikar S.S., Robinson J.R., Berndt S.I., Chan A.T., Figueiredo J.C., Lindor N.M., Song M., Schoen R.E. (2018). Leptin gene variants and colorectal cancer risk: Sex-specific associations. PLoS ONE.

[120] Wang D., Gao L., Gong K., Chai Q., Wang G. (2017). Increased serum leptin level in overweight patients with colon carcinoma: A cross-sectional and prospective study. Mol. Clin. Oncol..

[121] Zhou W., Tian Y., Gong H., Guo S., Luo C. (2014). Oncogenic role and therapeutic target of leptin signaling in colorectal cancer. Expert Opin. Ther. Targets.

[122] Greer K.B., Falk G.W., Bednarchik B., Li L., Chak A. (2015). Associations of srum adiponectin and leptin with Barrett’s Esophagus. Clin. Gastroenterol. Hepatol..

[123] Clemons N.J., Phillips W.A., Lord R.V. (2013). Signaling pathways in the molecular pathogenesis of adenocarcinomas of the esophagus and gastroesophageal junction. Cancer Biol. Ther..

[124] Wang Q.-Y., Shen Z.X. (2012). The expression and value of leptin and leptin receptor in human esophageal carcinoma. Lab. Med..

[125] Beales I.L.P., Ogunwobi O.O. (2021). Leptin activates Akt in oesophageal cancer cells via multiple atorvastatin-sensitive small GTPases. Mol. Cell. Biochem..

[126] Bain G.H., Collie-Duguid E., Murray G.I., Gilbert F.J., Denison A., Mckiddie F., Ahearn T., Fleming I., Leeds J., Phull P. (2015). Tumour expression of leptin is associated with chemotherapy resistance and therapy-independent prognosis in gastro-oesophageal adenocarcinomas. Br. J. Cancer.

[127] Ellis P.E., Barron G.A., Bermano G. (2020). Adipocytokines and their relationship to endometrial cancer risk: A systematic review and meta-analysis. Gynecol. Oncol..

[128] Wang S.-N., Lee K.-T., Ker C.-G. (2010). Leptin in hepatocellular carcinoma. World J. Gastroenterol..

[129] Ribatti D., Belloni A.S., Nico B., Di Comite M., Crivellato E., Vacca A. (2008). Leptin-leptin receptor are involved in angiogenesis in human hepatocellular carcinoma. Peptides.

[130] Zhang L., Yuan Q., Li M., Chai D., Deng W., Wang W. (2020). The association of leptin and adiponectin with hepatocellular carcinoma risk and prognosis: A combination of traditional, survival, and dose-response meta-analisys. BMC Cancer.

[131] Babic A., Bao Y., Qian Z.R., Yuan C., Giovannucci E.L., Aschard H., Kraft P., Amundadottir L., Stolzenberg-Solomon R., Morales-Oyarvide V. (2016). Pancreatic cancer risk associated with prediagnostic plasma levels of leptin and leptin receptor genetic polymorphisms. Cancer Res..

[132] Harbuzairu A., Oprea-Ilies G., González-Pérez R.R. (2018). Pancreatic cancer, leptin, and chemoresistance: Current challenges. Advances in Pancreatic Cancer.

[133] Liao L.M., Schwartz K., Pollak M., Graubard B.I., Li Z., Ruterbusch J., Rothman N., Davis F., Wacholder S., Colt J. (2013). Serum leptin and adiponectin levels and risk of renal cell carcinoma. Obesity (Silver Spring).

[134] Stattin P., Söderberg S., Hallmans G., Bylund A., Kaaks R., Stenman U.H., Bergh A., Olsson T. (2001). Leptin is associated with increased prostate cancer risk: A neste case-referent study. J. Clin. Endocrinol. Metab..

[135] López-Fontana C.M., Maselli M.E., Pérez-Elizalde R.F., Di Milta-Mónaco N.A., Uvilla-Recupero A.L., López-Laur J.D. (2011). Leptin increases prostate cancer aggressiveness. J. Physiol. Biochem..

[136] Alshaker H., Sacco K., Alfraidi A., Muhammad A., Winkler M., Pchejetski D. (2015). Leptin signalling, obesity and prostate cancer: Molecular and clinical perspective on the old dilemma. Oncotarget.

[137] Gorrab A., Pagano A., Ayed K., Chebil M., Derouiche A., Kovacic H., Gati A. (2020). Leptin promotes prostate cancer proliferation and migration by stimulating STAT3 pathway. Nutr. Cancer.

[138] Chen C., Chang Y.-C., Lan M.S., Breslin M. (2013). Leptin stimulates ovarian cancer cell growth and inhibits apoptosis by increasing cyclin D1 and Mcl-1 expression via the activation of the MEK/ERK1/2 and PI3K/Akt signaling pathways. Int. J. Oncol..

[139] Ghasemi A., Hashemy S.I., Aghaei M., Panjehpour M. (2018). Leptin induces matrix metalloproteinase 7 expression to promote ovarian cancer cell invasion by activating ERK and JNK pathways. J. Cell. Biochem..

[140] Ghasemi A., Saeidi J., Mohtashami M., Hashemy S.I. (2019). Estrogen-independent role of ERα in ovarian cancer progression induced by leptin/Ob-Rb axis. Mol. Cell. Biochem..

[141] Gislefoss R.E., Stenehjem J.S., Hektoen H.H., Andreassen B.K., Langseth H., Axcrona K., Weiderpass E., Mondul A., Robsahm T.E. (2018). Vitamin D, obesity and leptin in relation to bladder cancer incidence and survival: Prospective protocol study. BMJ Open.

[142] Kashiwagi E., Abe T., Kinoshita F., Ushijima M., Masaoka H., Shiota M., Netto G.J., Eto M., Mijamoto H. (2020). The role of adipocytokines and their receptors in bladder cancer: Expression of adiponectin or leptin is an independent prognosticator. Am. J. Transl. Res..

[143] Zou H., Liu Y., Wei D., Wang T., Wang K., Huang S., Liu L., Li Y., Ge J., Li X. (2016). Leptin promotes proliferation and metastasis of human gallbladder cancer through OB-Rb leptin receptor. Int. J. Oncol..

[144] Hanahan D., Weinberg R.A. (2011). Hallmarks of cancer: The next generation. Cell.

[145] Park J., Scherer P.E. (2011). Leptin and cancer: From cancer stem cells to metastasis. Endocr. Relat. Cancer.

[146] Myers M.G., Cowley M.A., Münzberg H. (2008). Mechanisms of leptin action and leptin resistance. Annu. Rev. Physiol..

[147] Dutta D., Ghosh S., Pandit K., Mukhopadhyay P., Chowdhury S. (2012). Leptin and cancer: Pathogenesis and modulation. Indian J. Endocrinol. Metab..

[148] Garofalo C., Surmacz E. (2006). Leptin and cancer. J. Cell Physiol..

[149] Andò S., Catalano S. (2011). The multifactorial role of leptin in driving the breast cancer microenvironment. Nat. Rev. Endocrinol..

[150] Feldman D.E., Chen C., Punj V., Tsukamoto H., Machida K. (2012). Pluripotency factor-mediated expression of the leptin receptor (OB-R) links obesity to oncogenesis through tumor-initiating stem cells. Proc. Natl. Acad. Sci. USA.

[151] Ghasemi A., Saeidi J., Azimi-Nejad M., Hashemy S.I. (2019). Leptin-induced signaling pathways in cancer cell migration and invasion. Cell. Oncol. (Dordr).

[152] Lennon H., Sperrin M., Badrick E., Renehan A. (2016). The obesity paradox in cancer: A review. Curr. Oncol. Rep..

[153] Lee D.H., Giovannucci E.L. (2019). The obesity paradox in cancer: Epidemiologic insight and perspectives. Curr. Nutr. Rep..

[154] Carbone S., Canada J.M., Billingsley H.E., Siddiqui M.S., Elagizi A., Lavie C.J. (2019). Obesity paradox in cardiovascular disease: Where do we stand?. Vasc. Health Risk Manag..

[155] Cespedes-Feliciano E.M., Kroenke C.H., Caan B.J. (2018). The Obesity Paradox in Cancer: How Important Is Muscle?. Annu. Rev. Nutr..

[156] Lam V.K., Bentzen S.M., Mohindra P., Nichols E.M., Bhooshan N., Vyfhuis M., Scilla K.A., Feigenberg S.J., Edelman M.J., Feliciano J.L. (2017). Obesity is associated with long-term improved survival in definitively treated locally advanced non-small cell lung cancer (NSCLC). Lung Cancer.

[157] Shepshelovich D., Xu W., Lu L., Fares A., Yang P., Christiani D., Zhang J., Shiraishi K., Ryan B.M., Chen C. (2019). Body Mass Index (BMI), BMI Change, and Overall Survival in Patients with SCLC and NSCLC: A Pooled Analysis of the International Lung Cancer Consortium. J. Thorac. Oncol..

[158] Ardesch F.H., Ruiter R., Mulder M., Lahousse L., Stricker B.H.C., Kiefte-de Jong J.C. (2020). The Obesity Paradox in Lung Cancer: Associations with Body Size Versus Body Shape. Front Oncol..

[159] Parker A.S., Lohse C.M., Cheville J.C., Thiel D.D., Leibovich B.C., Blute M.L. (2006). Greater body mass index is associated with better pathologic features and improved outcome among patients treated surgically for clear cell renal cell carcinoma. Urology.

[160] Waalkes S., Merseburger A.S., Kramer M.W., Herrmann T.R.W., Wegener G., Rusteimer J., Hofmann R., Schrader M., Kuczyk M.A., Schrader A.J. (2010). Obesity is associated with improved survival in patients with organ-confined clear-cell kidney cancer. Cancer Causes Control.

[161] Hakimi A.A., Furberg H., Zabor E.C., Jacobsen A., Schultz N., Ciriello G., Mikklineni N., Fiegoli B., Kim P.H., Voss M.H. (2013). An epidemiologic and genomic investigation into the obesity paradox in renal cell carcinoma. J. Natl. Cancer Inst..

[162] Albiges L., Hakimi A.A., Xie W., McKay R.R., Simantov R., Lin X., Lee J.-L., Rini B.I., Srinivas S., Bjarnason G.A. (2016). Body Mass Index and Metastatic Renal Cell Carcinoma: Clinical and Biological Correlations. J. Clin. Oncol..

[163] Navarro W.H., Loberiza F.R., Bajorunaite R., Van Besien K., Vose J.M., Lazarus H.M., Rizzo J.D. (2006). Effect of body mass index on mortality of patients with lymphoma undergoing autologous hematopoietic cell transplantation. Biol. Blood Marrow Transpl..

[164] Weiss L., Melchardt T., Habringer S., Boekstegers A., Hufnagl C., Neureiter D., Hopfinger G., Greil R., Egle A. (2014). Increased body mass index is associated with improved overall survival in diffuse large B-cell lymphoma. Ann Oncol..

[165] Stevenson J.K.R., Qiao Y., Chan K.K.W., Beca J., Isaranuwatchai W., Guo H., Schwartz D., Arias J., Gavura S., Dai W.F. (2019). Improved survival in overweight and obese patients with aggressive B-cell lymphoma treated with rituximab-containing chemotherapy for curative intent. Leuk. Lymphoma.

[166] McQuade J.L., Daniel C.R., Hess K.R., Mak C., Wang D.Y., Rai R.R., Park J.J., Haydu L.E., Spencer C., Wongchenko M. (2018). Association of body-mass index and outcomes in patients with metastatic melanoma treated with targeted therapy, immunotherapy, or chemotherapy: A retrospective, multicohort analysis. Lancet Oncol..

[167] Smith L.K., Arabi S., Lelliot E.J., McArthur G.A., Sheppard K.E. (2020). Obesity and the impact on cutaneous melanoma: Friend or foe?. Cancer (Basel).

[168] Brunner A.M., Sadrzadeh H., Feng Y., Drapkin B.J., Ballen K.K., Attar E.C., Amrein P.C., McAfee S.L., Chen Y.B., Neuberg D.S. (2013). Association between baseline body mass index and overall survival among patients over age 60 with acute myeloid leukemia. Am. J. Hematol..

[169] Hines R.B., Shanmugam C., Waterbor J.W., McGwin G., Funkhouser E., Coffey C.S., Posey J., Manne U. (2009). Effect of comorbidity and body mass index on the survival of African-American and Caucasian patients with colon cancer. Cancer.

[170] Schlesinger S., Siegert S., Koch M., Walter J., Heits N., Hinz S., Jacobs G., Hampe J., Schafmayer C., Nöthlings U. (2014). Postdiagnosis body mass index and risk of mortality in colorectal cancer survivors: A prospective study and meta-analysis. Cancer Causes Control.

[171] Amptoulach S., Gross G., Kalaitzakis E. (2015). Differential impact of obesity and diabetes mellitus on survival after liver resection for colorectal cancer metastases. J. Surg. Res..

[172] Shahjehan F., Merchea A., Cochuyt J.J., Li Z., Colibaseanu D.T., Kasi P.M. (2018). Body Mass Index and Long-Term Outcomes in Patients with Colorectal Cancer. Front. Oncol..

[173] Tran C.G., Hill E.E., Jensen B., Stark A.C., Flannery M., Berg D.J., Chan C.H.F. (2019). Survival benefit of obesity in stage IV colorectal cancer: Better tolerability of chemotherapy?. J. Clin. Oncol..

[174] Tsang N.M., Pai P.C., Chuang C.C., Chuang W.C., Tseng C.K., Chang K.P., Yen T.C., Lin J.D., Chang J.T.C. (2016). Overweight and obesity predict better overall survival rates in cancer patients with distant metastases. Cancer Med..

[175] Petrelli F., Cortellini A., Indini A., Tomasello G., Ghidini M., Nigro O., Salati M., Dottorini L., Iaculli A., Varricchio A. (2020). Obesity paradox in patients with cancer: A systematic review and meta-analysis of 6,320,365 patients. MedRxiv.

[176] Mantzoros C.S., Bolhke K., Moschos S., Cramer D.W. (1999). Leptin in relation to carcinoma in situ of the breast: A study of pre-menopausal cases and controls. Int. J. Cancer.

[177] Petridou E., Papadiamantis Y., Markopoulos C., Spanos E., Dessypris N., Trichopoulos D. (2000). Leptin and insulin growth factor I in relation to breast cancer (Greece). Cancer Causes Control.

[178] Paik S.S., Jang S.-M., Jang K.S., Lee K.H., Choi D.C., Jang S.J. (2009). Leptin expression correlates with favorable clinicopathologic phenotype and better prognosis in colorectal adenocarcinoma. Ann. Surg. Oncol..

[179] Thompson K.J., Lau K.N., Johnson S., Martinie J.B., Iannitti D.A., McKillop I.H., Sindram D. (2011). Leptin inhibits hepatocellular carcinoma proliferation via p38-MAPK-dependent signalling. HPB (Oxford).

[180] Dalamaga M., Migdalis I., Fargnoli J.L., Papadavid E., Bloom E., Mitsiades N., Karmaniolas K., Pelecanos N., Tseleni-Balafouta S., Dionyssiou-Asteriou A. (2009). Pancreatic cancer expresses adiponectin receptors and is associated with hypoleptinemia and hyperadiponectinemia: A case-control study. Cancer Causes Control.

[181] Colakoglu M.K., Bostanci E.B., Ozdemir Y., Dalgic T., Aksoy E., Ozer I., Ozogul Y., Oter V. (2017). Roles of adiponectin and leptin as diagnostic markers in pancreatic cancer. Bratisl. Lek. Listy..

[182] Rivadeneira D.B., DePeaux K., Wang Y., Kulkarni A., Tabib T., Menk A.V., Sampath P., Lafyatis R., Ferris R.L., Sarkar S.N. (2019). Oncolytic Viruses Engineered to Enforce Leptin Expression Reprogram Tumor-Infiltrating T Cell Metabolism and Promote Tumor Clearance. Immunity.

[183] Araújo-Vilar D., Santini F. (2019). Diagnosis and treatment of lipodystrophy: A step-by-step approach. J. Endocrinol. Investig..

[184] Paz-Filho G., Mastronardi C.A., Licinio J. (2015). Leptin treatment: Facts and expectations. Metabolism.

[185] Rodriguez A.J., Mastronardi C.A., Paz-Filho G.J. (2015). New advances in the treatment of generalized lipodystrophy: Role of metreleptin. Ther. Clin. Risk Manag..

[186] Friedman J. (2016). The long road to leptin. J. Clin. Investig..

[187] Levin B.E., Lutz T.A. (2017). Amylin and Leptin: Co-Regulators of Energy Homeostasis and Neuronal Development. Trends Endocrinol. Metab..

[188] Cauchard S., Bermudez-Humaran L.G., Blugeon S., Laugier C., Langella P., Cauchard J. (2011). Mucosal co-immunization of mice with recombinant lactococci secreting VapA antigen and leptin elicits a protective immune response against Rhodococcus equi infection. Vaccine.

[189] Waldman A.D., Fritz J.M., Lenardo M.J. (2020). A guide to cancer immunotherapy: From T cell basic science to clinical practice. Nat. Rev. Immunol..

[190] De la Cruz-Merino L., Palazón-Carrión N., Henao-Carrasco F., Nogales-Fernández E., Álamo-de la Gala M., Vallejo-Benítez A., Chiesa M., Sánchez-Margalet V., GEICAM (Spanish Breast Cancer Research Group), GÉTICA (Spanish Group for Cancer Immuno-Biotherapy) (2019). New horizons in breast cancer: The promise of immunotherapy. Clin. Transl. Oncol..

[191] Vera F., Pino J., Campos-Cabaleiro V., Ruiz-Fernandez C., Mera A., Gonzalez-Gay M.A., Gomez R., Gualillo O. (2018). Obesity, Fat Mass and Immune System: Role for Leptin. Front. Physiol..

[192] Hu W., Wang G., Huang D., Sui M., Xu Y. (2019). Cancer Immunotherapy Based on Natural Killer Cells: Current Progress and New Opportunities. Front. Immunol..

[193] Bernotiene E., Palmer G., Gabay C. (2006). The role of leptin in innate and adaptive immune responses. Arthritis Res. Ther..

[194] Chen D.S., Mellman I. (2013). Oncology meets immunology: The cancer-immunity cycle. Immunity.

[195] Faroogi I.S., Matarese G., Lord G.M., Keogh J.M., Lawrence E., Agwu C., Sanna V., Jebb S.A., Perna F., Fontana S. (2002). Beneficial effects of leptin on obesity, T cell hyporesponsiveness, and neuroendocrine/metabolic dysfunction of human congenital leptin deficiency. J. Clin. Investig..

[196] Naylor C., Petri Jr W.A. (2016). Leptin regulation of immune responses. Trends Mol. Med..

[197] Sanchez-Margalet V., Barco-Sanchez A., Vilarino-Garcia T., Jimenez-Cortegana C., Perez-Perez A., Henao-Carrasco F., Virizuela-Echaburu J.A., Nogales-Fernandez E., Alamo-de la Gala M.C., Lobo-Acosta M.A. (2019). Circulating regulatory T cells from Breast cancer patients in response to neoadjuvant chemotherapy. Transl. Cancer Res..

[198] Toker A., Nguyen L.T., Stone S.C., Yang S.Y.C., Katz S.R., Shaw P.A., Clarke B.A., Ghazarian D., Al-Habeeb A., Easson A. (2018). Regulatory T Cells in Ovarian Cancer Are Characterized by a Highly Activated Phenotype Distinct from that in Melanoma. Clin. Cancer Res..

[199] Flamminger A., Weisbach L., Huland H., Tennstedt P., Simon R., Minner S., Bokemeyer C., Sauter G., Schlomm T., Trepel M. (2013). High tissue density of FOXP3+ T cells is associated with clinical outcome in prostate cancer. Eur. J. Cancer.

[200] Woo E.Y., Yeh H., Chu C.S., Schlienger K., Carroll R.G., Riley J.L., Kaiser L.R., June C.H. (2002). Cutting edge: Regulatory T cells from lung cancer patients directly inhibit autologous T cell proliferation. J. Immunol..

[201] Avgerinos K.I., Spyrou N., Mantzoros C.S., Dalamaga M. (2019). Obesity and cancer risk: Emerging biological mechanisms and perspectives. Metabolism.

[202] Zorena K., Jachimowicz-Duda O., Slezak D., Robakowska M., Mrugacz M. (2020). Adipokines and obesity. Potential link to metabolic disorders and chronic complications. Int. J. Mol. Sci..

